# Point-prevalence survey of hospital acquired infections in three acute care hospitals in Northern Nigeria

**DOI:** 10.1186/s13756-020-00722-9

**Published:** 2020-05-11

**Authors:** Usman Abubakar

**Affiliations:** Pharmacy Department, Ibrahim Badamasi Babangida (IBB) Specialist Hospital, Minna, Niger State, Nigeria

**Keywords:** Hospital acquired infection, Point-prevalence, Nigeria

## Abstract

**Background:**

Effective infection prevention and control strategies require reliable data describing the epidemiology of hospital acquired infections (HAIs), and this is currently lacking in Nigeria. The objective of this study was to evaluate the prevalence, types and risk factors associated with HAIs in acute care hospitals in Northern Nigeria.

**Methods:**

A pilot point-prevalence survey was conducted in three acute care hospitals in Northern Nigeria between April and May 2019 using a protocol developed by the European Centre for Disease Prevention and Control. Patients admitted into the wards at or before 8.00 am on the survey date were included. Patients’ medical records were reviewed by a clinical pharmacist with the support of the attending physician and nurse to identify HAIs.

**Results:**

Of the 321 patients surveyed, 50 HAIs were identified among 46 patients translating into a point-prevalence of 14.3%. The most common HAIs were bloodstream infection (38.0%), surgical site infections (32.0%) and pneumonia (12.0%). Neonatal (53.0%), pediatric surgical (26.7%) and surgical (10.1%) specialties had the highest prevalence. Device associated infections represented 16% of all HAIs including bloodstream infections and pneumonia. Of all the HAIs, 15 (30.0%) were present at the time of admission while 75.5% originated from the current hospitals. Univariate analysis showed that newborn (less than 1 month old) (OR: 4.687 95% CI: 1.298–16.927), intubation (OR: 3.966, 95% CI: 1.698–9.261), and neonatal (OR: 41.538 95% CI: 4.980–346.5) and pediatric surgical (OR: 13.091 95% CI: 1.532–111.874) specialties were significantly associated with HAI.

**Conclusion:**

The prevalence of HAI was relatively high compared to other developing countries and was significantly associated with neonatal and pediatric surgical specialties. Hospital infection control strategies should be strengthened to reduce the burden of HAIs.

## Background

Healthcare Associated Infection (HAI) is a public health problem affecting hospitalized patients and it is associated with morbidity, mortality, and healthcare costs [1–3]. HAIs can be associated with antibiotic resistant pathogens which make treatment more difficult and expensive. In Europe, about one-third of micro-organisms isolated in HAI are resistant to antibiotics [4]. In Africa, higher rates of antibiotic resistant (10–100%) are reported among isolates from HAIs [5, 6]. Available evidence shows that the prevalence and types of HAI varies among countries with lower rates observed in developed countries. In the United States (US), one in 24 hospitalized patients develops HAI with high rates for pneumonia, surgical site infection (SSI) and gastro-intestinal infections [2]. In Europe, the prevalence of HAI in acute care settings is 6.5% (range 5.4–7.8%). Pneumonia and other lower respiratory tract infections, urinary tract infection, and SSI are the most common HAI in European acute care hospitals [4]. Approximately 16% of hospitalized patients in developing countries are diagnosed with HAI [7]. The high rate of HAI in developing countries is attributed to inadequate infection control practices owing to the lack of infection control policy and guideline, and the dearth of infection control health professionals [8]. Other factors include lack of infrastructure, inconsistent surveillance [9], overcrowding, scarcity of resources [10], poor sanitation and poor management of hospital waste [11].

HAIs can be prevented through implementation of infection control and prevention program, surveillance of HAIs, proper waste management and proper training of hospital staff on biosafety [12]. An effective infection prevention and control program requires active surveillance to generate data that would describe the prevalence and risk factors associated with HAI. In addition, surveillance data is needed to measure the impact of infection control and prevention programs and also to prioritize areas for further interventions and resource allocation. The European Center for Disease Prevention and Control (ECDC) and the National Health Safety Network (NHSN) provide continuous surveillance for HAI and antibiotic consumption in Europe and the US respectively. In Nigeria, there is currently no surveillance system to provide estimates of HAI in acute care hospitals. Previous estimates of HAI have reported prevalence rates between 2.5 and 6.3% [6, 13, 14]. However, the studies used single center and retrospective design as against prospective active surveillance, which is the gold standard [15]. Prospective active surveillance is capital intensive and time-consuming. However, point-prevalence survey is a valid and reliable alternative to prospective active surveillance to provide estimates of HAIs [16]. The former method is more cost-effective than the latter and could be conducted with few surveyors. Therefore, point-prevalence study is suitable for use to estimate the burden of HAI in hospitals with limited resources. The objective of this pilot point-prevalence study was to determine the point-prevalence and risk factors associated with HAI in acute care hospitals.

## Method

### Study design and setting

This point-prevalence survey was conducted in 3 acute care hospitals located in two states in Northern Nigeria using a protocol adapted from the point prevalence survey of healthcare-associated infections and antimicrobial use in European acute care hospitals protocol version 5.3 [17]. The survey was conducted in one public university teaching (approximately 560 beds) and two public general (200 and 100 beds respectively) hospitals. The selected hospitals provide acute care services including internal medicine, surgery, obstetrics and gynecology, pediatrics, neonatal care, psychiatry, intensive care and ear, nose and throat care. The wards in each specialty in the selected hospitals were divided into male and female wards, and each ward was further subdivided into ‘A’ and ‘B’ respectively. None of the wards had more than 30 patients on admission at the time of the survey. This arrangement made the survey of all patients in one ward in a single day feasible.

### Inclusion and exclusion criteria

All units/departments in the selected hospitals were surveyed with the exception of outpatient, adult accident and emergency, and psychiatric units. Emergency pediatric unit was included because some patients are monitored for > 24 h. It is important to note that two of the hospitals had an intensive care unit at the time of this study. However, there was no patient on admission on the day the unit was scheduled for survey. The neonatal unit in the selected hospitals is divided into two subunits: in-born (for neonates born in the hospital) and out-born (for neonates born at home or at other healthcare facilities) wings, and both wings were included in the survey. All patients admitted into the ward and monitored for more than 24 h in the participating hospitals were included in the survey. Patients who were in the ward before or at 8.00 a.m. and were not discharged at the time of the survey were included. Daycare patients, those transferred out of the wards before the survey were excluded.

### Data collection

Information including patient's age, gender, dates of admission and survey, surgery since admission, presence of any invasive device (peripheral and central vascular catheters, urinary catheter and intubation), McCabe score, patient/consultant specialty, presence of HAI and the results of microbiological investigations were collected using a data collection form. Information was obtained by reviewing the medical and nursing records of the surveyed patients and identified HAIs were discussed with the attending physician and nurse. Data was collected by a clinical pharmacist who has expertise in infectious diseases and experience in reviewing patient chart/record. All the patients in a single ward were surveyed on the same day. Data collection lasted for 2 weeks in one of the hospitals. The survey was conducted from 22 April to 24 May 2019.

### Outcome measures

Hospital acquired infection and device-associated HAIs were defined based on the criteria described in the point prevalence survey of healthcare-associated infections and antimicrobial use in European acute care hospitals Protocol version 5.3 [17]. Asymptomatic bacteriuria and ophthalmic neonatorum were not considered as HAIs. The following criteria were used to determine device-associated HAI: i) presence of urinary catheter at least 7 days before the onset of urinary tract infection, ii) central and peripheral vascular catheter was in place at least 48 h before onset of bloodstream infection, and iii) intubation in the preceding 48 h before the onset of pneumonia.

### Data analysis

Data was entered into, double checked for coding errors, cleansed and analyzed using Statistical Package for the Social Sciences (SPSS) version 23. Categorical data was reported as frequency and percentage while continuous data was presented as mean (standard deviation) or median. Risk factors for HAI were determined using bivariate and multivariate regression analysis. Only variables that demonstrated statistical significance (*P* < 0.05) in the bivariate analyses were included in the multivariate model.

## Results

### Hospital characteristics

Three public hospitals participated in this pilot point-prevalence survey including one university teaching hospital and two general hospitals. The total bed capacity of the selected hospitals was approximately 860 beds. A total of 321 hospitalized patients were included in this pilot survey representing 37.3% of the total bed space in the hospitals. There was an active infection control and prevention (IPC) team in two of the hospitals, however, none of the hospitals had an active surveillance program at the time of the survey. All the hospitals had a microbiology department with a functional laboratory to process specimens which was opened on every day of the week. However, lack of steady electricity supply affects the processing of specimens collected for microbiological culture and sensitivity test.

### Patient characteristics

Three hundred and 21 hospitalized patients were included in the analysis with a median age of 27 years (range: 1–93 years). The median length of stay on the survey date was 8 days (IQR; 1–231 days). Females constituted approximately 58% of all patients surveyed and about one-third of the patients had surgery during the current admission. On the day of the survey, central catheter, peripheral catheter, urinary catheter and intubation was present in 2.8, 54.2, 15.0 and 8.7% of the surveyed patients, respectively. Table 1 describes the clinical and demographic characteristics of the surveyed patients.

**Table 1:** Hospital and clinical characteristics of the patients surveyed

Variable	Frequency	Percentage
Median age (IQR)	27 (1–93)	
Median length of hospital stay (IQR)	8 (1–231)	
Gender		
Male	129	40.2
Female	186	57.9
Surgery since admission	93	29.0
McCabe score		
Non-fatal	228	71.0
Ultimately fatal	7	2.2
Rapidly fatal	31	9.7
Unknown	19	6.7
Missing	36	11.2
Central catheter present on the survey date	9	2.8
Peripheral catheter present on the survey date	174	54.2
Urinary catheter present on the survey date	48	15.0
Intubation on the survey date	28	8.7
Type of Hospital		
Secondary	136	42.4
Tertiary	185	57.6
Specialty		
Pediatric medical	37	11.5
Neonatal	28	8.7
Medical	87	27.1
Surgical	69	21.5
Obstetrics and gynecology	70	21.8
Pediatric surgical	30	9.3

### Prevalence of hospital acquired infections

A total of 50 HAIs were identified in 46 patients translating into a point-prevalence of 14.3%. Forty-two (91.3%) patients had one HAI while the remaining four (8.7%) patients had 2 HAIs each. The prevalence of HAI in the tertiary hospital (15.1%) was comparable to the prevalence in secondary hospitals (13.2%). The prevalence of HAIs ranged from 0.0 to 20.0% in the selected hospitals. The prevalence HAI was significantly higher in the neonatal unit (53.6%) followed by pediatric surgical (26.7%), surgical (10.1%) and OBG (10.0%) units. About one-third of all HAIs were detected in the neonatal unit while 18% each were identified in medical and pediatric surgical units. Table 2 shows the prevalence of HAI among the patients disaggregated based on the patient/specialty.

**Table 2:** Prevalence of HAI among the surveyed patients by patient’s specialty

Specialty	Prevalence of HAI among surveyed patients N (%)	Prevalence among all HAI N (%)
Pediatric medical	1 (2.7)	2 (4.00)
Neonatal	15 (53.6)	15 (30.0)
Medical	8 (9.2)	9 (18.0)
Surgical	7 (10.1)	8 (16.0)
Obstetrics and gynecology	7 (10.0)	7 (14.0)
Pediatric surgical	8 (26.7)	9 (18.0)
Total	47 (14.6)	50 (100.0)

### Types of hospital acquired infections

Bloodstream infections; including neonatal sepsis and neonatal laboratory confirmed bloodstream infection (5.9%) and surgical site infection (5.0%) were the most common HAI and represented 38.0 and 32.0% of all HAI respectively. The prevalence of pneumonia was 1.9% and it constituted 12.0% of all the HAIs. Other types of HAI include gastrointestinal infections (10.0%), EENT (6.0%) and SST (1.9%). Table 3 shows the distribution of the types of HAIs based on patient’s specialty.

**Table 3:** Distribution of HAI based on patient’s specialty

HAI	Prevalence among survey patients	Prevalence among all HAI	Prevalence N (%)					
			Pediatric medical	Neonatal	Medical	Surgical	Obstetrics and gynecology	Pediatric surgical
BSI	19 (5.9)	19 (38.0)	–	15 (100.0)	1 (11.1)	–	1 (14.3)	2 (22.2)
SSI	16 (5.0)	16 (32.0)	–	–	–	4 (50.0)	6 (85.7)	6 (66.7)
Pneumonia	6 (1.9)	6 (12.0)	–	–	4 (44.4)	2 (25.0)	–	–
GI	5 (1.6)	5 (10.0)	–	–	2 (22.2)	2 (25.0)	–	1 (11.1)
EENTI	2 (0.6)	3 (6.0)	2 (100.0)	0 (0.0)	1 (11.1)	–	–	–
SSTI	1 (0.3)	1 (2.0)	–	–	1 (11.1)	–	–	–

*BSI* Bloodstream infection, *SSI* Surgical Site Infections, *GI* Gastrointestinal infection, *EENTI* Eye, Ear, Nose and Throat Infection, *SSTI* Skin and Soft Tissue Infection

### Device associated infections and micro-organisms

Of the HAI identified, 16% (8 infections) were device-associated infections. Overall, 26.3% (*N* = 5/19) of the patients with bloodstream infections had central or peripheral catheter present 48 h before the onset of the infection. Devices-associated pneumonia occurred in 33.3% (*N* = 2/6) of the cases while one patient who had urinary tract infection within 30 days after surgery had urinary catheter present in the 7 days preceding the infection. Overall, 15 (30.0%) HAIs were present at the time of admission and consisted of 10 neonatal sepsis, two bloodstream infection, two SSI, and one neonatal LCBI. Most of the HAI (75.5%) originated from the current hospital while 18.9 and 5.6% were from other hospitals and other/unknown origin, respectively. Micro-organisms were isolated in four HAIs including *Staphylococcus aureus* (sensitive to gentamicin, erythromycin, streptomycin, ciprofloxacin, levofloxacin, rifampicin, amoxicillin and norfloxacin; and resistant to cotrimoxazole), Methicillin resistant *Stapyhlococcus aureus* (sensitive to gentamicin, chloramphenicol, and clindamycin; and resistant to ciprofloxacin), *Escherichia coli* (sensitive to gentamicin, perfloxacin, streptomycin, and ofloxacin; and resistant to amoxicillin, ciprofloxacin, chloramphenicol, sparfloxacin, cotrimoxazole and amoxicillin-clavulanic acid) and *Proteus species* (sensitive to ceftriaxone, ciprofloxacin and imipenem; and resistant to amoxicillin, levofloxacin and cefixime).

### Factors associated with hospital acquired infections

Univariate logistic regression analysis showed that newborn (less than 1 month) (OR: 4.687, 95% CI: 1.298–16.927), intubation during hospitalization (OR: 3.966, 95% CI: 1.698–9.261) and patients in neonatal (OR: 41.538, 95% CI: 4.980–346.500) and pediatric surgical (OR: 13.091, 95% CI: 1.532–111.874) specialties were significantly associated with HAI. There was no association between HAI and gender, duration of stay and surgery since admission. Table 4 summarizes the factors associated with HAI. There was no significant association between HAI and the variables in the multivariate regression model.

**Table 4:** Univariate logistic regression analysis of factors associated with HAI

Variable	Odds ratio	95% CI	***P*** value
**Age group**			
**< 1 month**	**4.687**	**1.298–16.927**	**0.018**
1 month – 18 years	0.692	0.192–2.490	0.573
18–65 years	0.439	0.133–1.451	0.177
> 65 years	Reference	–	–
Gender	0.807	0.425–1.533	0.513
Surgery since admission	0.583	0.305–1.116	0.104
McCabe score			
Non-fatal	Reference	–	–
Ultimately fatal	1.021	0.119–8.761	0.985
Rapidly fatal	0.656	0.188–2.286	0.508
Unknown	1.633	0.510–5.234	0.409
Any invasive device	0.634	0.324–1.240	0.183
Central catheter present	1.740	0.350–8.50	0.498
Peripheral catheter present	0.909	0.486–1.699	0.765
Urinary catheter present	2.022	0.945–4.327	0.070
**Intubation**	**3.966**	**1.698–9.261**	**0.001**
**Specialty**			
Pediatric medical	Reference	–	–
**Neonatal**	**41.538**	**4.980–346.500**	**0.001**
Medical	3.646	0.439–30.247	0.231
Surgical	4.065	0.481–34.379	0.198
Obstetrics and gynecology	4.000	0.473–33.826	0.203
**Pediatric surgical**	**13.091**	**1.532–111.874**	**0.019**
Duration of stay before the date of the survey	1.007	0.995–1.019	0.261
Type of hospital			
Secondary care hospital	Reference	–	–
Tertiary care hospital	1.169	0.617–2.214	0.631

Of the 321 patients surveyed, 257 (80.1%) used at least one antibiotic on the day of the survey. The indications for antibiotic use included: community acquired infection (38.7%), surgical antibiotic prophylaxis (22.5%), hospital acquired infection (16.3%), medical prophylaxis (14.9%) and unknown (7.6%). The most common antibiotics used were metronidazole (30.5%), ciprofloxacin (17.1%), ceftriaxone (16.8%), amoxicillin-clavulanic acid combination (12.5) and gentamicin (11.8%). The data for antimicrobial use among the patients has been published elsewhere [18].

## Discussion

The current study revealed that the point-prevalence of HAI was 14.3% with bloodstream and surgical site infections accounting for two-third of all the HAIs. Neonatal and pediatric surgical specialties had the highest prevalence and represented more than 50% of all the HAI. The prevalence of HAI in the present study was higher than the 2.6–6.3% reported in previous retrospective studies conducted in Nigeria [6, 13, 14]. However, the current study is comparable to the cumulative prevalence of HAI in developing countries [7] and Ethiopia [19], but higher than the prevalence in Ghana [20], South Africa [21], Iran [22], the United States [2] and the European hospitals [4]. It is important to note that methodological differences between these studies influence the findings and could explain the inconsistencies. Although overcrowding is not an issue in the selected hospitals, the rate of HAIs was high. This could be attributed to the lack of a national infection control policy and guideline, poor infection control practices, lack of surveillance, inadequate clinical waste management and the dearth of infection control personnel in the hospitals. Bloodstream infections, particularly neonatal sepsis, surgical site infections and pneumonia were the most common HAIs in the current study, consistent with the findings of an Iranian study [22], but inconsistent with the results in other countries [2, 4]. Previous studies conducted in Nigeria demonstrated that urinary tract infection (UTI) was the most common HAI [6, 13]. In contrast, the current study did not identify any UTI among the hospitalized patients surveyed and this could be explained by the requirement to perform microbiological investigation before diagnosing hospital acquired UTI which was not frequently observed. Intravascular catheterization is an independent risk factor for hospital acquired bloodstream infections [22].

High rate of surgical site infections among the surveyed patients was consistent with the result of a previous study conducted in South-Western Nigeria [13]. These infections are preventable with adequate infection control, blood glucose control and surgical antibiotic prophylaxis. However, compliance with surgical antibiotic prophylaxis recommendations in Nigeria is inadequate [23]. Therefore, strategies to improve compliance with infection control and surgical antibiotic prophylaxis, particularly the use of antimicrobial stewardship interventions, are recommended. Antimicrobial stewardship interventions were effective in improving compliance with antibiotic selection, timing and duration of surgical antimicrobial prophylaxis in a recent study conducted in Nigeria [24]. Of the surveyed patients, newborn (less than 1 month old), those with intubation tube and those admitted under neonatal and pediatric surgical specialties had significantly higher Odds for HAIs. This was consistent with previous studies for age [15] and intubation [22, 25], respectively. The high risk of HAIs among newborns depict suboptimal levels of intrapartum and post-delivery care, particularly among neonates born outside the hospital.

The current study is the first prospective point-prevalence study to describe the prevalence of HAI in Northern Nigeria. There are some limitations in the study, thus, the results should be interpreted with caution. First, the findings cannot be generalized for the entire region and Nigeria at large because the data was collected in three hospitals only. Secondly, the study design used (point-prevalence survey) which is not the gold standard for the surveillance of HAI and the prevalence reported could be either over- or under-estimated. However, point-prevalence study is a valid method for the surveillance of HAIs in resource limited settings [16]. Thirdly, there was no concurrent validation of the collected data during the survey due to lack of resources and as a result, the findings could be over- or under-estimated. Fourthly, information regarding the number of laboratory- or radiologically-confirmed HAIs as well as indicators of laboratory capacity and hospital infection control were not collected. Future studies should include multiple centers and addressed the limitations highlighted in this study. Despite the limitations, the current study provides a glimpse of the prevalence of HAIs in Northern Nigeria.

## Conclusion

The prevalence of HAIs is relatively high compared to other developing countries with bloodstream and surgical site infections as well as pneumonia being the most common. Acquiring HAI was significantly associated with newborn, intubation and neonatal and pediatric surgical specialties. Hospital infection control and prevention strategies need to be strengthened to improve the quality of care among hospitalized patients.

## Data Availability

All data generated or analyzed during this study are included in this published article.

## Abbreviations

HAI: Hospital acquired infections
US: The United States
ECDC: European Center for Disease Prevention and Control
NHSN: National Health Safety Network
IPC: Infection Control and Prevention
BSI: Bloodstream Infection
SSI: Surgical Site Infections
GI: Gastrointestinal Infection
EENTI: Eye, Ear, Nose and Throat Infection
SSTI: Skin and Soft Tissue Infection
LCBI: Laboratory Confirmed Bloodstream Infection
